# Imaging evaluation of uterine arteries in potential living donors for uterus transplantation: a comparative study of MRA, CTA, and DSA

**DOI:** 10.1007/s00330-021-08350-6

**Published:** 2021-11-12

**Authors:** Henrik Leonhardt, Anne Thilander-Klang, John Båth, Marit Johannesson, Niclas Kvarnström, Pernilla Dahm-Kähler, Mats Brännström

**Affiliations:** 1 Department of Radiology, Institute of Clinical Sciences, Sahlgrenska University Hospital, Sahlgrenska Academy at University of Gothenburg, Bruna stråket 11B, SE-413 45, Gothenburg, Sweden; 2 Department of Radiation Physics, Institute of Clinical Sciences, Sahlgrenska Academy at University of Gothenburg, Gothenburg, Sweden; 3 Department of Transplantation, Institute of Clinical Sciences, Sahlgrenska Academy at University of Gothenburg, Gothenburg, Sweden; 4 Department of Obstetrics and Gynecology, Institute of Clinical Sciences, Sahlgrenska Academy at University of Gothenburg, Gothenburg, Sweden

**Keywords:** Computed tomography angiography, Digital subtraction angiography, Living donors, Magnetic resonance angiography, Uterus

## Abstract

**Objective:**

To evaluate uterine arteries (UA) of potential living donors for uterus transplantation (UTx) by comparison of CT angiography (CTA), digital subtraction angiography (DSA), and MR angiography (MRA) with care taken to minimize radiation doses.

**Methods:**

Prospective donors for a clinical UTx trial were included. CTA, DSA, and MRA measurements in three predefined segments of the UAs were evaluated. Radiation doses were estimated and 1-year graft survival was recorded.

**Results:**

Twelve potential donors (age 37–62 years) were investigated. There was no difference in visualized average UA lumen diameter when comparing CTA (mean 2.0 mm, SD 0.4), DSA (mean 2.1 mm, SD 0.6), and MRA (mean 2.0 mm, SD 0.3). MRA was not able to fully evaluate 10 (43%) out of 23 UA that proved to be patent on DSA. One UA was not identified by any of the modalities, and three MRA-absent UAs were identified by both CTA and DSA. The estimated mean effective dose was lower for DSA (5.1 mSv, SD 2.8) than CTA (7.1 mSv, SD 2.0), but not significantly (*p* value = 0.06). Three potential donors were excluded due to UA pathology and one due to adenomyosis. Eight donors underwent hysterectomy, with 1-year graft survival in six women.

**Conclusion:**

MRI including MRA should be the initial modality to examine potential UTx donors to acquire valuable details of uterine anatomy, and if UAs are fully visualized, there is no need for further angiographic methods with radiation. If UAs are not visualized by MRA, CTA may be performed and in selective cases with addition of the invasive modality DSA.

**Key Points:**

• *For uterine transplantation, pelvic MRI with MRA provides information of the uterine structure and of the diameters of uterine arteries in living donors.*

• *Failure of MRA to demonstrate uterine arteries could be followed by CTA which will visualize the uterine arteries in a majority of cases. If MRA and additional CTA provide inconclusive results, the uterine arteries should be further evaluated by DSA.*

• *Information of CTA can be used in the angio-system for DSA settings to minimize the radiation and contrast media doses.*

**Supplementary Information:**

The online version contains supplementary material available at 10.1007/s00330-021-08350-6.

## Introduction

Uterus transplantation (UTx) is the first available treatment, although still at a clinical experimental stage, for absolute uterine factor infertility (AUFI), a condition caused either by a uterine absence or presence of a nonfunctional uterus. The prevalence of AUFI in the UK has been estimated to 12,000 women of fertile age [1].

The proof of concept of UTx as a treatment for AUFI came with the first live birth after UTx in 2014 [2], as part of nine living donor (LD) UTx procedures within the world’s first UTx trial [3]. Several more live births have followed from this first UTx trial [4, 5], as well as from other LD UTx trials elsewhere [4, 6–8].

It is proposed that one of several possible reasons for early graft failure, with thrombosis of uterine vessels, is related to poor uterine artery (UA) quality and secondary hypo-perfusion of the uterus after transplantation [8, 9]. The proportion of early graft failures in our initial UTx study from 2013 was 2/9, and in that trial, only MRI was used for evaluating the uterus and its vasculature prior to donor inclusion [3]. It is noteworthy that the uterus is normally supplied by blood flow from three bilateral arteries (uterine, ovarian, vaginal), but after transplantation, only the two UAs will remain as feeding vessels. The donors of the two failures in our initial study were both > 60 years of age and postmenopausal [3]. At reperfusion, the measured blood flow in these two cases was lower than in most other cases. The cause of failures was most likely related to UA atherosclerosis, with the extents influenced by high donor age and postmenopausal state [10]. A relation between graft failure and higher LD age was also seen in a UTx study in the USA [9]. We stipulate that some cases of graft loss may occur due to inadequate postprocedural arterial blood flow to the uterus secondary to small diameters of the lumina of the UAs.

The role of imaging in the evaluation of LDs is to identify abnormalities of the uterus or vasculature that could pose a potential problem for UTx. It is reasonable to perform imaging as safe as possible in this relatively young healthy population with donation by altruistic reason. Exposure to radiation should obviously be kept at a minimum. To date, there are no studies showing which imaging modality is the best choice for evaluating the UAs in LDs.

The aim of the present study was to compare contrast-enhanced CT angiography (CTA), digital subtraction angiography (DSA), and magnetic resonance angiography (MRA) to evaluate LD UAs before donation, with care taken to minimize radiation doses. This included a strategy to use the CTA information to plan and optimize the DSA procedure.

## Materials and methods

### Ethics and patients

This prospective comparative study was part of a clinical trial (NCT02987023) of robotic surgery for LDs of UTx [11], approved by the Regional Ethics Committee (362–16) with written informed consents from all participants. Inclusion criteria for potential donors were as follows: family/close friend to recipient, age < 65 years, no serious medical/psychological comorbidity, present non-smoking, and previous normal obstetric history, including live birth(s). Imaging findings on the initial screening MRI that would exclude use of the uterus included the following: intramural leiomyoma of size > 4 cm, intracavitary/submucosal and unresectable polyp/leiomyoma, extensive diffuse or large (> 2 cm) focal adenomyosis, uterine malformation, and/or thin uterine scar after cesarean section. Exclusion criteria based on UA imaging results were the following: absence of bilateral patent UA, diameter of UA of < 1 mm at one (women ≥ 50 years of age) or more than one (women < 50 years of age) of the measured locations (see below), and/or presence of atherosclerotic plaques/caliber variations. For postmenopausal women, estrogen-containing hormone replacement therapy was recommended from initial inclusion in the study, which was typically 10 months before UTx.

### Imaging modalities and technique

After an initial clinical examination, including transvaginal ultrasound, the protocol consisted of contrast-enhanced pelvic CTA in the arterial phase, conventional DSA focusing on the internal iliac arteries with the UAs, and pelvic MRI including dynamic contrast-enhanced MRA of the pelvic vessels. Details of these examinations are presented in Table 1.

**Table 1:** Imaging parameters and protocol details in living donor candidate evaluation

Imaging	Technical features	Protocol parameters
**CTA** pelvic arteries from the trochanter minor to the pelvic crest	Scanner: Somatom Force (Siemens Healthineers)	Care kV, semi-reference 100 kV Reference mAs: 289 mAs Rotation time: 0.5 s Pitch: 0.8 Single collimation of 0.6 mm and with a total collimation of 57.6 mm
	Intravenous contrast medium: low osmolarity nonionic iodinated (Omnipaque®, GE Healthcare) 350 mg I/ml	500 mg I/kg, 25 g I/kg/s, injection time 20 s, followed by 40 ml saline Scan in the arterial phase using bolus triggering, ROI placed in the distal abdominal aorta, scan start at 150 HU plus in ROI with 20 s scan delay
	Reconstruction	Reconstruction kernel: Bf36 Iterative reconstruction: Admire strength 4 FOV: 360–400 mm Axial: 0.6 mm and 1.5 mm MPR: 4 mm, interval 3 mm in coronal and sagittal planes MIP: 7 mm, interval 2 mm in axial and coronal planes
**DSA** internal iliac arteries focusing on the uterine arteries	Artis-Q ceiling (Siemens Healthineers)	CT-guided angulation of the C-arm (RAO or LAO)
	Intravenous contrast medium: low osmolarity nonionic iodinated (Omnipaque®, GE Healthcare) 240 mg I/ml	Injection of approximatively 10 ml, 3–5 ml/s, through a 4-Fr hydrophilic cobra-shaped catheter (Impress®, Merit Medical) placed in the internal iliac artery shortly above the orifice of the uterine artery, 1–2 image series at each side, initially 4 images/s (4/2/1) after fluoroscopic-driven selection of exposure parameters
**MRA** pelvic vessels	3T Achieva dStream (Philips Medical Systems)	Coronal 3D T1W gradient echo (FFE) sequence FOV: 337 × 262 mm^2^ Acq. voxel: 0.99 × 1.1 × 3 mm^3^ (Rec. voxel: 0.6 × 0.6 × 1.5 mm^3^) Acq. matrix: 340 × 239 TR/TE, shortest (approx. 5/1.79 ms) Flip angle: 30° Acquisition time: 24 s
	Intravenous contrast medium: Gadoterate meglumine (Dotarem®, Gothia Medical) 279.3 mg/ml or gadoteric acid (Clariscan®, GE Healthcare) 0.5 mmol/ml	20 ml, 2 ml/s Scanned in arterial phase using bolus track Second (venous) phase acquired 20 s after arterial phase
	Reconstruction	Radial MIP tumble images, 15 projections with a radial angle of 12° around both the RL and FH axes

Abbreviations: *CTA* computed tomography angiography, *DSA* digital subtraction angiography, *MRA* magnetic resonance angiography, *FOV* field of view, *ROI* region of interest, *HU* Hounsfield units, *MPR* multiplanar reformation, *MIP* maximal intensity projection, *RAO* right anterior oblique, *LAO* left anterior oblique, *FFE* fast field echo, *TR* repetition time, *TE* echo time, *RL* right–left, *FH* feet–head

**Fig. 1:**
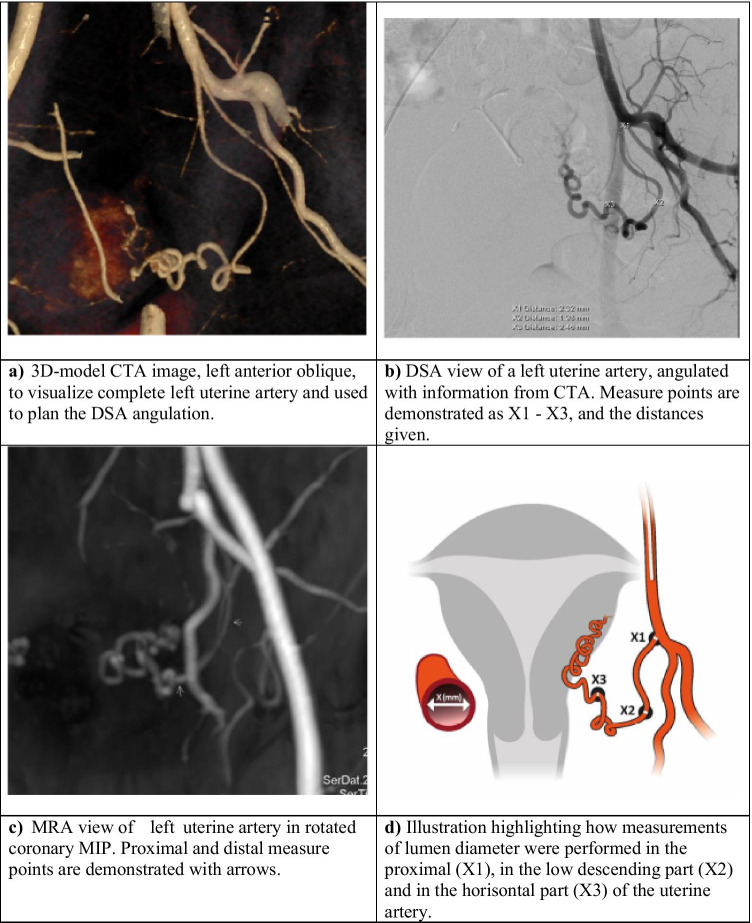
Example of the reproduction of the uterine artery in an oblique plane in the different imaging modalities, (**a**) CTA, (**b**) DSA, (**c**) MRA, and the location of the measurement points are given in **d**. Abbreviations: CTA = computed tomography angiography, DSA = digital subtraction angiography, MRA = magnetic resonance angiography, and MIP = maximal intensity projection

Potential donors underwent CTA and MRI/MRA on the same day, except for two (cases #3 and #4), where MRI/MRA was performed approximatively 1 month before CTA–DSA. In eleven of the twelve potential donors, the CTA was used to plan for the DSA procedure, usually performed the day after CTA. The previously acquired CTA was imported into the workstation of the angiographic suite (syngo.via workplace, Siemens Healthineers). At the start of the DSA, two perpendicular fluoroscopic images centered over the pelvic region were saved and a 2D/3D registration was performed using the CTA volume. The internal iliac artery orifice was marked with a circle in the CT volume, and that circle was displayed as an overlay on the fluoroscopic image during the procedure to guide the diagnostic catheter. This was part of an effort to reduce both procedure and fluoroscopic time. To further minimize radiation exposure during angiography, a 3D model of the CT volume was displayed on the angio-system’s secondary screen (Fig. 1b). A projection correspondent to the C-arm angulation enabled optimal angulation for visualization of the entire UA, sometimes partly obscured by other branches from the internal iliac artery. The DSA procedure was standardized and performed by one radiologist (J.B.) with 2 years’ experience of interventional radiology (IR), under initial supervision by a senior radiologist (H.L.) with > 25 years’ experience of IR. After ultrasound-guided puncture of the common femoral artery, an 11-cm 4-Fr sheath (Super Sheath, Boston Medical) was inserted. To catheterize the internal iliac artery ipsilateral and contralateral over the aortic bifurcation, a 4-Fr glidecatheter (IMPRESS®Cobra1, Merit Medical) and a glidewire (0.035 Terumo Glidewire, Terumo) were used. The catheter was placed in the internal iliac artery proximal to any major branches before angiographic images were exposed. Care was taken to avoid catheterization of the uterine arteries because an adverse dissection would be perceived disastrous to the donor and recipient, however clinically probably not significant. The angiographic exams were ended with retraction of the 4-Fr sheet followed by external compression.

### Arterial evaluation and measurements

**Fig. 2:**
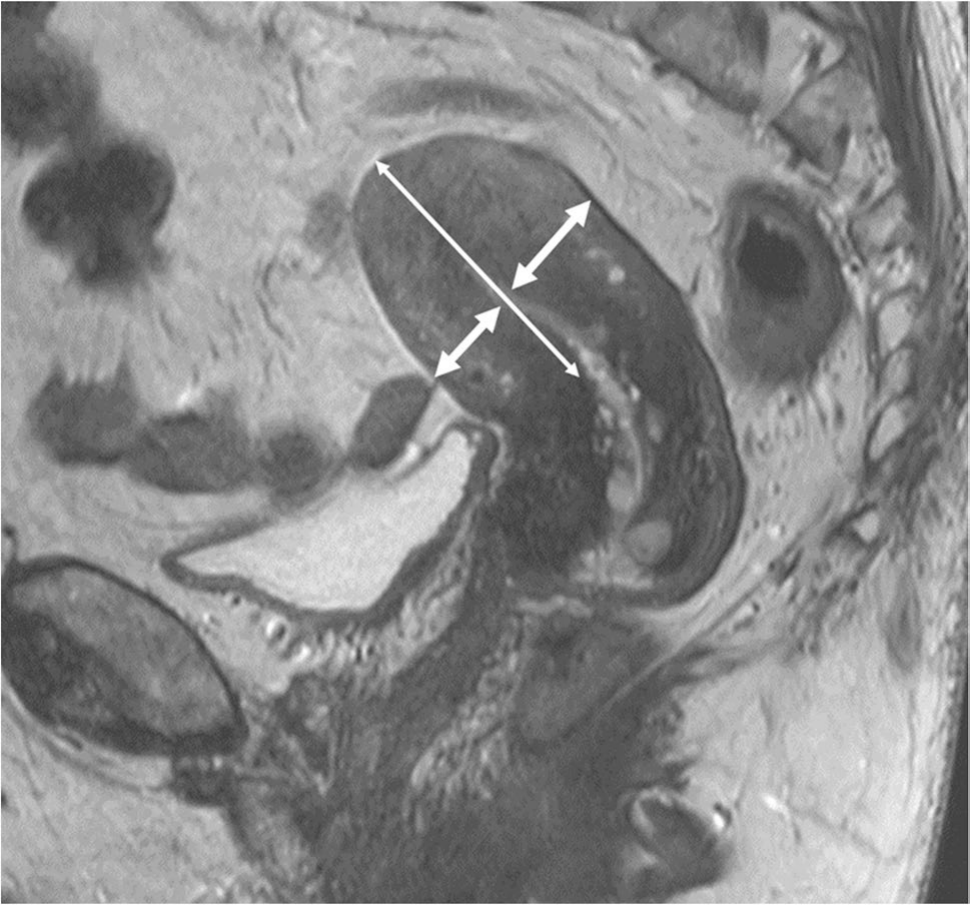
T2-wieghted sagittal image through the uterine midline in a potential donor illustrating size assessment. Thick arrows demonstrate the measurements performed on myometrial thickness. The mean of the measurement on the anterior versus the posterior wall of the uterine body was used as the parameter *myometrial thickness*. The long thin arrow demonstrates the parameter *length fundus*–*isthmus*

One radiologist (J.B.) analyzed the UAs from the CTA, DSA, and MRA examinations and performed the measurements of the lumen (Fig. 1). The subjects were anonymized, and the measurements were performed at random, blinded to patient characteristics. One senior radiologist (H.L.) evaluated the MRI/MRA, including measurements of uterine body length (fundus–isthmus) and myometrial thickness (Fig. 2).

Measurements were done in three predefined segments of the UA: proximally, in the low descending part, and in the horizontal part (Fig. 1b, d). Care was taken to identify and measure the visually narrowest part of the UA in each of the segments. The MRA arterial measurements were performed on post-contrast maximal intensity projections (MIP). The CTA measurements were assessed predominantly on the axial thin (0.6 mm) slices. The DSA measurements were assessed on selected DSA images, with optimal view.

### Dosimetry

The radiation burden to the potential donor was estimated both as the effective dose (*E*) to the whole body (in mSv) and as the uterine absorbed dose (*D*_uterine_, in mGy). For the estimation of *E*, two different Monte Carlo–based software programs were used: for CTA, the CT-Expo 2.5 [12], and for DSA, the PCXMC 2.0 ROT [13].

In the PCXMC software, the possibility to scale the female phantom according to the patient’s height and weight was used. The exposure parameters, tube voltage, beam filtration, field size, projection angle, focus-rotation distance, and the given dose–area product (DAP) value, were used as input for the estimation of *E* for each one of the projection angles used at the DSA examination. As the DAP value from the fluoroscopic irradiations only was given as a total by the DSA system, it was divided into equal parts and added to the DAP value from the exposure for each projection angle. The number of projections used and their projection angles differ among the examinations due to individual adaptation.

In the CT-Expo software, one cannot scale the female phantom by size or weight. Exposure parameters, CT model and used tube voltage, beam filtration, tube current, rotation time, total beam collimation, and pitch, together with the scan length and the location of the irradiation on the “body,” were used as input for the estimation of *E*.

The tissue-weighting factors given in ICRP publication 103 [14] were used. The mean organ absorbed dose to the uterus (*D*_uterine_) was also estimated in the two Monte Carlo software programs used, as it is one of the 13 remainder tissues included in the estimation of *E*.

### Statistics

The pairwise comparisons (CTA–DSA, MRA–DSA, MRA–CTA) of the measured dimensions of the UA lumen were performed using both Bland–Altman analysis and Pearson’s correlation coefficient. Non-detectable lumen was excluded in the calculation of Pearson’s correlation coefficient. The mean value and standard deviations of *E* and *D*_uterine_ were determined for the 12 cases.

## Results

The general characteristics of the 12 included women (aged 37–62, median 51 years old) are given in Table 2. The postmenopausal women that subsequently underwent donor hysterectomy either were already on hormone replacement therapy (HRT) since the age of menopause (case #1) or started HRT at initial inclusion in the study (cases #2, #6, #8). Results of general uterine morphology by MRI, inclusion/exclusion, and 1-year graft outcome are given in Table 3.

**Table 2:** Patient characteristics of potential donors for UTx

Case	Age (years)	BMI (kg/m^2^)	Relationship to recipient	Previous smoker	Menopausal state
1	62	24.7	Mother	No	Post
2	61	23.6	Mother-in-law	No	Post
3	52	21.7	Mother	No	Pre
4	48	21.8	Mother	No	Pre
5	51	23.1	Mother-in-law	Yes	Pre
6	57	30.5	Mother	No	Post
7	61	28.2	Mother	No	Post
8	55	24.8	Mother	Yes	Post
9	48	24.2	Friend	No	Pre
10	45	26.6	Mother	No	Pre
11	46	22.5	Mother	No	Pre
12	37	22.0	Sister	No	Pre

Abbreviations: *BMI* body mass index, *UTx* uterus transplantation

**Table 3:** Diagnostic uterus outcome of potential donors and 1-year graft survival

Case	Length fundus–isthmus (mm)	Myometrial thickness (mm)	MRI-detected uterine morphology	Accepted for UTx	Rejected for UTx due to	1-year graft survival
1	62	19	Subserosal myoma/focal adenomyosis (< 2 cm)	Yes	–	Yes
*2*	*31*	*15*	*Normal*	*No*	*UA arteriosclerosis*	*–*
*3*	*73*	*23*	*Normal*	*No*	*UA absence unilateral*	*–*
4	51	22	Multiple subserosal/intramural myomas (1.5 cm)	Yes	–	Yes
*5*	*58*	*25*	*Submucosal and intramural myomas/diffuse adenomyosis*	*No*	*UA occlusion*	*–*
6	86	25	Intracavitary myoma (5 cm)	Yes	–	Yes
*7*	*69*	*21*	*Focal adenomyosis (3.5 cm)*	*No*	*Uterine pathology*	*–*
8	50	18	Intramural myoma (1 cm)	Yes	–	No; ischemia hysterectomy 6 months post UTx
9	54	19	Normal	Yes	–	Yes
10	63	18	Normal	Yes	–	Yes
11	35	18	Normal	Yes	–	No; ischemia hysterectomy 1 month post UTx
12	65	23	Normal	Yes	–	Yes

The four cases not accepted for donation are in italics. In case #6, a pedunculated myoma was hysteroscopically resected before she was accepted

Abbreviations: *UTx* uterus transplantation, *UA* uterine artery/arterial

### Uterine pathology and artery assessments

**Fig. 3:**
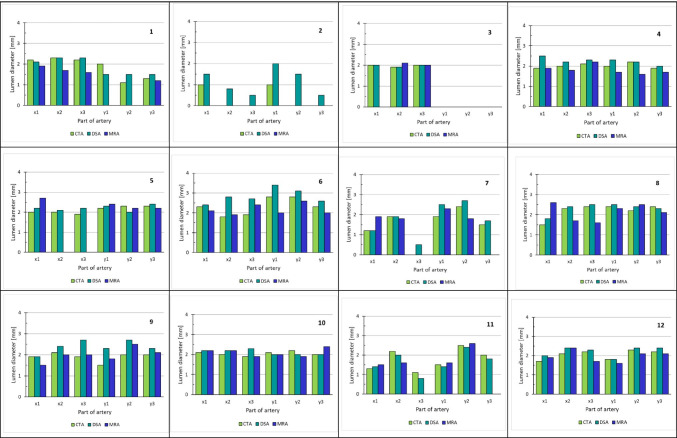
The measured lumen diameter in millimeters at three different parts of the uterine artery in the images received from different angiography examinations in CTA, DSA, and MRA are given for each one of the 12 potential donors. Cases #2, #3, #5, and #7 were the ones not included for uterus transplantation. *x* and *y* indicate the right and the left uterine artery, respectively. 1 indicates the proximal part, 2 the low descending part, and 3 the horizontal part of the uterine artery; see Fig. 1d. Abbreviations: CTA = computed tomography angiography, DSA = digital subtraction angiography, MRA = magnetic resonance angiography, and MIP = maximal intensity projection

All 12 women underwent imaging by CTA, DSA, and MRI/MRA (Fig. 1a–c). MRI-detected uterine pathology, i.e., leiomyomas and/or adenomyosis, were found in six cases (Table 2). Apart from stenoses, no other uterine arterial pathologies such as aneurysms or dissection were found. Results concerning lumen diameters at the three specific locations (see Fig. 1d) are given in Fig. 3 and Table 4.

**Table 4:** The measured lumen diameters in millimeters at three different parts of the uterine artery in the images received from different angiography examinations in CTA, DSA, and MRA are given for the 12 potential donors

Case	Position	CTA (mm)	DSA (mm)	MRA (mm)
**1**	x1	2.2	2.1	1.9
	x2	2.3	2.3	1.7
	x3	2.2	2.3	1.6
	y1	2	1.5	
	y2	1.1	1.5	
	y3	1.3	1.5	1.2
*2*	x1	1	1.5	
	x2		0.8	
	x3		0.5	
	y1	1	2	
	y2		1.5	
	y3		0.5	
*3*	x1	2	2	
	x2	1.9	1.9	2.1
	x3	2	2	2
	y1			
	y2			
	y3			
**4**	x1	1.9	2.5	1.9
	x2	2	2.2	1.8
	x3	2.1	2.3	2.2
	y1	2	2.3	1.7
	y2	2.2	2.2	1.6
	y3	1.9	2	1.7
*5*	x1	2	2.2	2.7
	x2	2	2.1	
	x3	1.9	2.2	
	y1	2.2	2.3	2.4
	y2	2.3	2	2.2
	y3	2.3	2.4	2.2
**6**	x1	2.3	2.4	2.1
	x2	1.8	2.8	1.9
	x3	1.9	2.7	2.4
	y1	2.8	3.4	2
	y2	2.8	3.1	2.6
	y3	2.3	2.6	2
*7*	x1	1.2	1.2	1.9
	x2	1.9	1.9	1.8
	x3		0.5	
	y1	1.9	2.5	2.3
	y2	2.4	2.7	1.8
	y3	1.5	1.7	
**8**	x1	1.5	1.8	2.6
	x2	2.3	2.4	1.7
	x3	2.4	2.5	1.6
	y1	2.4	2.5	2.3
	y2	2.2	2.4	2.5
	y3	2.4	2.3	2.1
**9**	x1	1.9	1.9	1.5
	x2	2.1	2.4	2
	x3	1.9	2.7	2
	y1	1.5	2.3	1.8
	y2	2	2.7	2.5
	y3	2	2.3	2.1
**10**	x1	2.1	2.2	2.2
	x2	2	2.2	2.2
	x3	1.9	2.3	1.9
	y1	2.1	2	2
	y2	2.2	2	1.9
	y3	2	2	2.4
**11**	x1	1.3	1.4	1.5
	x2	2.2	2	1.6
	x3	1.1	0.8	
	y1	1.5	1.4	1.6
	y2	2.5	2.4	2.6
	y3	2	1.8	
**12**	x1	1.7	2	1.9
	x2	2.1	2.4	2.4
	x3	2.2	2.3	1.7
	y1	1.8	1.8	1.6
	y2	2.3	2.4	2.1
	y3	2.2	2.4	2.1

No data—indicates no visibility of that part of the artery. Cases #2, #3, #5, and #7 (in italics) were the ones not included for uterus transplantation. x and y indicate the right and the left uterine artery, respectively. 1 indicates the proximal part, 2 the low descending part, and 3 the horizontal part of the uterine artery; see Fig. 1d. Abbreviations: *CTA* computed tomography angiography, *DSA* digital subtraction angiography, *MRA* magnetic resonance angiography

In only one case (#3) was one UA not identified by any of the three angiographic methods. UAs with completely measurable lumina were not detected in 10/23 (43%) and 4/23 (17%) DSA-verified open arteries by MRA and CTA, respectively. One of these arteries (case #1, left side) was barely visible on MRA and questionable and generally thin on CTA, but DSA visualized it as proximally divided into two small-caliber UA branches. This donor was accepted for surgery. There were no significant differences in visualized average arterial lumen diameter determined by CTA (mean 2.0 mm, SD 0.4) compared with DSA (mean 2.1 mm, SD 0.6) and MRA (mean 2.0 mm, SD 0.3).

**Fig. 4:**
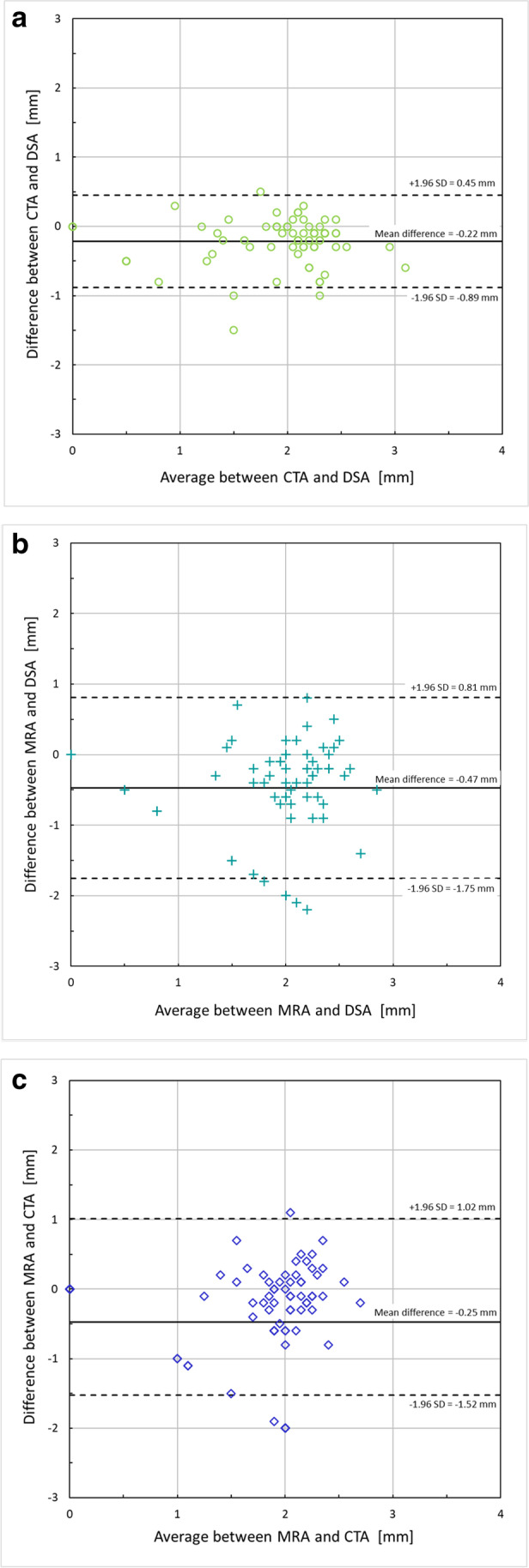
**a** The difference in the measured lumen diameters between CTA and DSA is given as a function of the mean value of the measured lumen diameters in CTA and DSA. The solid line indicates the mean value of the difference in lumen diameters and the dotted lines indicate the corresponding upper and lower confidence levels. **b** The difference in the measured lumen diameters between MRA and DSA is given as a function of the mean value of the measured lumen diameters in MRA and DSA. The solid line indicates the mean value of the difference in lumen diameters and the dotted lines indicate the corresponding upper and lower confidence levels. **c** The difference in the measured lumen diameters between MRA and CTA is given as a function of the mean value of the measured lumen diameters in MRA and CTA. The solid line indicates the mean value of the difference in lumen diameters and the dotted lines indicate the corresponding upper and lower confidence levels. Abbreviations: CTA = computed tomography angiography, DSA = digital subtraction angiography, MRA = magnetic resonance angiography, and MIP = maximal intensity projection

The highest correlation (Supplementary Figure 1a-b) of measured diameters was between CTA–DSA (Pearson’s *r* =  + 0.74), with lower correlations between MRA–DSA (*r* =  + 0.38) and MRA–CTA (*r* =  + 0.31). The agreement in the measured lumen diameters was also analyzed using Bland–Altman analysis where a mean difference in the lumen diameters is given as well as the confidence interval (CI) of 95% agreement (± 1.96 SD) (Fig. 4). The best agreement was found between CTA–DSA (mean difference =  − 0.22 mm, CI (− 0.89 to 0.45 mm)), the lowest between MRA–DSA (mean difference =  − 0.47 mm, CI (− 1.75 to 0.81 mm)), and for MRA–CTA (mean difference =  − 0.25 mm, CI (− 1. 52 to 1.02 mm)).

In seven out of twelve women, the image-fusion technique, as described above, enabled use of only one DSA series bilaterally to receive sufficient information of the UA.

### Dosimetry

**Fig. 5:**
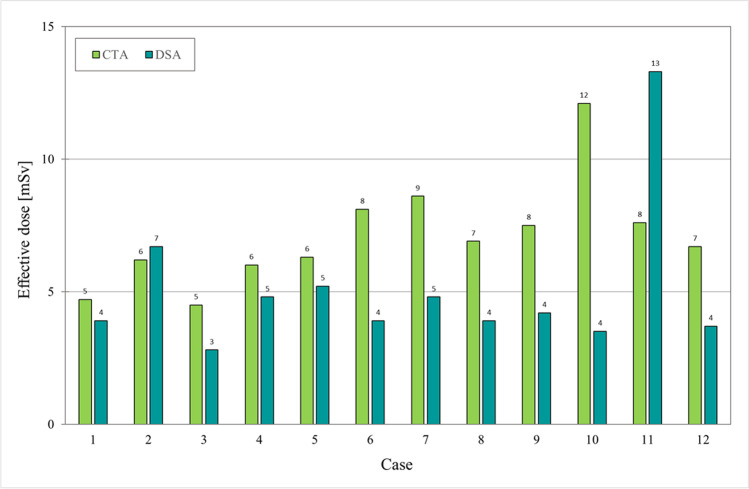
Estimated effective dose (in mSv) given for the CTA (lime) and DSA (teal) examinations, respectively, for each one of the 12 potential donors. Cases #2, #3, #5, and #7 were the ones not accepted for UTx. Abbreviations: CTA = computed tomography angiography and DSA = digital subtraction angiography

**Fig. 6:**
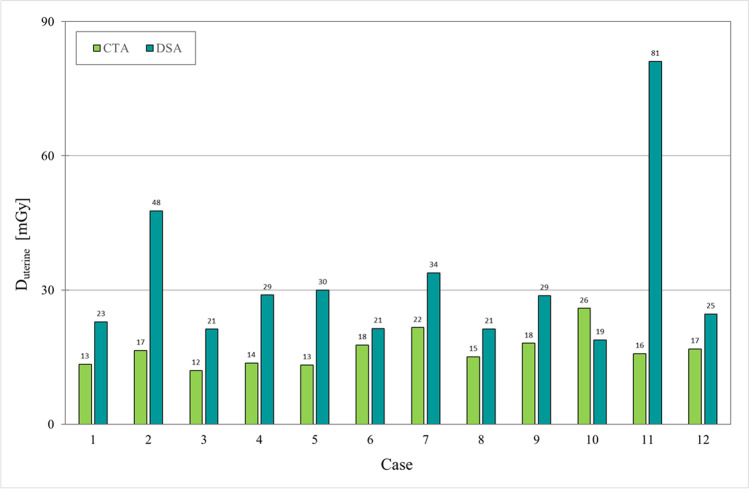
Estimated uterine absorbed dose, *D*_uterine_ (in mGy), given for the CTA (lime) and DSA (teal) examinations, respectively, for each one of the 12 potential donors. Cases #2, #3, #5, and #7 were the ones not accepted for UTx. Abbreviations: CTA = computed tomography angiography and DSA = digital subtraction angiography

For each donor, the estimated *E* and the *D*_uterine_ for CTA and DSA are presented in Figs. 5 and 6. The estimated mean value of *E* was lower for the DSA procedures (mean 5.1 mSv, SD 2.8) than the CTA procedures (mean 7.1 mSv, SD 2.0), but not significantly (*p* value = 0.06). The mean value of *D*_uterine_ for DSA was estimated to 32 mGy, SD 17.4, and for CTA. 17 mGy, SD 3.9 (*p* value = 0.02). In case #2, the outlier value of 47.7 mGy for *D*_uterine_ at DSA was most probably due to this woman’s hip prosthesis (the only one with such device in the cohort). The outlier value of 13.3 mSv for *E* (and the corresponding *D*_uterine_ = 81 mGy) at DSA in case #11 was due to an unintentional use of the medium-dose exposure program opposing the default low-dose exposure program in all other donors. In case #10, the outlier value of 12.1 mSv for *E* (and the corresponding *D*_uterine_ = 25.9 mGy) at CTA was due to the body composition with a wide lower abdomen causing the automatic exposure to increase the used mAs value to 378, compared to the set value of 289, to be able to fulfill the image quality criteria set. The same applies for case #7, but here, the mAs value was 325.

### Uterus transplantation and clinical outcome

Out of the twelve potential donors, eight donors were accepted for surgery and underwent donor hysterectomy. Three women were excluded according to the exclusion criteria due to non-acceptable UAs bilaterally (Table 3). One potential donor uterus (case #7) was excluded due to extensive adenomyosis detected on MRI. One uterus (case #6) showed a 5-cm pedunculated leiomyoma within an expanded uterine cavity. After hysteroscopy and successful resection of the leiomyoma, a normalized uterus was shown on MRI and the woman was accepted for donation.

Eight UTx procedures were performed, and two grafts were removed in the interval of 1–4 months post UTx, due to suboptimal blood perfusion of uteri with signs of endometrial degeneration in both cases (Table 3). The suboptimal blood perfusion was visualized as color-Doppler blood flow only in the peripheral uterus and not in the center, as well as patchy necrosis at cervical biopsies. Concerning the two donors, where hysterectomy was performed post UTx due to hypo-perfusion, one uterus from a 55-year-old postmenopausal (also previous smoker) donor (case #8) had acceptable DSA-measured UA lumen size with minimum 1.8 mm at any of the measurement locations (Table 4). The other uterus from a 46-year-old pre-menopausal, never-smoking donor (case #11) had a minimum lumen size of 0.8 mm, but the other lumen locations were of acceptable sizes, according to the predefined inclusion criteria.

The total 1-year graft survival was 6/8 (75%) (Table 3). Interim results are to date that 4/6 have ongoing pregnancy/live birth and 2/6 are continuing with embryo transfers. All non-pregnant women have spontaneous, regular menstruations as a sign of graft functionality.

## Discussion

Uterus transplantation is a new fertility treatment and the only fertility option for thousands of women with AUF [2]. One proposed mechanism for early graft failure is ischemia, most likely secondary poor quality of the feeding UAs. In the present study, alternatives for preoperative assessment of UAs in potential LDs for UTx were investigated. We found that MRA is a feasible first modality to evaluate for structural uterine abnormalities that may preclude transplantation and that MRA sequences can be added to these protocols. This will give information needed in the majority of cases. When UAs are not properly imaged by MRA, CTA will visualize the UAs in a majority and should be the second modality due to its non-invasiveness. In cases of inconclusive results on MRA and CTA, evaluation by DSA, optimized by CTA data, can be applied.

We present the first comparative study on different imaging modalities, including the gold standard DSA, to investigate UAs before uterus donation. In the first UTx trial, the uterine imaging of LDs was performed solely with ultrasound and MRI/MRA, focusing on the uterine structure and veins [2, 15]. However, we procured good lengths of deep uterine veins bilaterally in all cases, despite anatomical variations, and did not encounter a relationship between uterine veins and graft outcome. Instead, low perfusion at back-table preparations and high donor age seemed to be related to graft failure [2, 3]. Thus, one focus of the present trial was to evaluate the UAs in all potential donors, with preset inclusion criteria where the threshold of the UA diameter was set to around 1 mm.

Our results show that, when visualized, the measured UA diameter is similar with MRA, CTA, or DSA, with the highest correlation between CTA and DSA. We found a wide variation in the DSA-visualized diameters from 0.5 to 3.4 mm (mean 2.1 mm). For comparison, there exists one other study on UTx donors using CTA for imaging of UAs, and in some cases also combined with MRA, reporting UA diameters of means 2.1 (1–5) mm and 2.2 (1–3) mm by CTA and MRA, respectively [16]. Somewhat larger measurements were found in a recent study of four UTx donors with mean 2.9 (2.5–3.5) mm [17]. Notably, these two studies [16, 17] were not designed for comparative imaging analysis, although one study [16] suggests that MRA and CTA may have complimentary roles. It is questionable whether an additional radiation dose of dual-phase CT angiograms could be justified for uterus LDs in the future, based on the results of the present study, where we received sufficient CTA information by examining only pelvic arteries in single phase, with minimal radiation dose.

In the evaluation of donors, also the non-vascular part of the uterus should be evaluated to exclude pathological changes which may negatively influence the fertility potential. A combination of ultrasound and MRI will be able to accurately assess the uterus and also visualize uterine vessels, with variations existing concerning UAs [18]. However, because of high spatial resolution, CTA can be advantageous to clarify complex vascular anatomy before transplantation [19], and to detect calcified plaques.

In the present study, we used selective DSA, which is the gold standard in visualizing small-caliber arteries, but novel CT techniques may challenge [20]. It is noteworthy that DSA is invasive with risks for the patient such as hematoma/dissection, but such adverse events did not occur in this study. Our strategy to fuse pre-interventional CT volumes with the angio-system for localization and to minimize radiation was successful.

We found an incidence of stenosed arteries, rendering exclusion according to predefined criteria, of 3/12. The high incidence is possibly attributable to the relatively high age, with associated long time of a hypoestrogenism which will initiate UA atherosclerosis [10]. However, a postmenopausal woman may still have adequate UAs if she has been on estrogens since menopause, as demonstrated by live births from uteri of ages 61–62 years at donation [2, 21]. In the trial from the USA, the mean age of the 18 LDs was 38 years [22], as compared to 52 years in the present study. Noteworthy is that a branched, double, and thin UA may still qualify for UTx, as shown in case #1.

Although we had strict inclusion criteria concerning UA diameters to ensure reasonable blood flow, the failure rate was as high as 2/8. The two uteri were removed within the first months because of clear signs of hypo-perfusion. Since there were no signs of endometrial regeneration, there was no potential for future embryo implantation. It may well be that the inclusion criteria should be modified, based on the experience from these two cases and future studies. One suggestion could be that the minimal size of the UAs for inclusion should be somewhat increased and should possibly be even greater in a previous smoker than in a never-smoker.

Comparing radiation doses is challenging and needs specification as the mean value of effective dose was lower in DSA (5.1 mSv) compared to CTA (7.1 mSv), while the opposite is valid when comparing the organ dose, *D*_uterine_, where the DSA gave almost twice the dose (32 mGy) as the corresponding in CTA (17 mGy). The much higher standard deviation in DSA is partly due to the outlier in case #11, but also an indication of the influence the operator has as well as the patient size.

One limitation of the study is the small number of patients, which may affect the generalizability. However, the present study is the most comprehensive study in this new field since we used three imaging modalities, including DSA. Another limitation is that data of pregnancy and live births are not fully reported, although we report that 4/6 of the patients have ongoing pregnancy/live birth. The remaining two patients are undergoing repeated embryo transfer attempts, and it is quite likely that at least one of these women will achieve pregnancy, based on experience from normal in vitro fertilization, showing a cumulative live birth rate of above 70% after six IVF cycles with their associated multiple embryo transfers [23].

The finding that the performance of MRA is inferior to that of CTA and DSA is somewhat unexpected. We did not find any specific factor, such as motion artifacts or inadequate timing of contrast bolus, explaining why some of the UAs in our cohort were not fully visualized by MRA. However, most of these arteries were of small caliber according to DSA and probably below the limit of spatial resolution of detection for these particular cases in our MRA protocol. The MRA technique may be improved by imaging one UA (left–right) at a time, by displaying smaller field of views and voxel sizes, or by injection of a higher dose of the contrast agent. These issues could be evaluated in future studies.

Based on the findings of the present study, we propose, in LD UTx evaluation, to rely on MRA findings of UA patency and diameters if satisfactory. When MRA fails to visualize a patent UA, CTA should be used, and in a few still unclear cases, DSA is needed.

## Supplementary Information

Below is the link to the electronic supplementary material.

Supplementary file1 (DOCX 103 KB)

## Abbreviations

AUFI: Absolute uterine factor infertility
CI: Confidence interval
CTA: Computed tomography angiography
*D*: Absorbed dose (unit mGy)
DAP: Dose–area product (unit mGycm^2^)
DSA: Digital subtraction angiography
*D*_uterine_: Uterine absorbed dose (unit mGy)
*E*: Effective dose (unit mSv)
Gy: Gray (unit of absorbed dose)
HRT: Hormone replacement therapy
ICRP: International Commission on Radiological Protection
IR: Interventional radiology
IVF: In vitro fertilization
LD: Living donor
MIP: Maximal intensity projection
MPR: Multiplanar reformation
MRA: Magnetic resonance angiography
SD: Standard deviation
Sv: Sievert (unit of effective dose)
UA: Uterine artery
UTx: Uterus transplantation

## Declarations

### Guarantor

The scientific guarantor of this publication is Dr. Henrik Leonhardt, MD, PhD.

### Statistics and biometry

One of the authors has significant statistical expertise.

### Study subjects or cohorts overlap

As part of a clinical trial (NCT02987023), we have reported on a sub-cohort of 8 donors and the recipients included in the current study but with emphasis on the surgical procedure, in two publications (ref 11 and 21) from 2020. In the present study, we report on additional 4 women (thus in total 12 cases) with emphasis on imaging in the evaluation of potential living donors for uterus transplantation and 1-year graft survival.

### Methodology

• prospective

• comparative diagnostic and prognostic study

• performed at one institution
