# Supplementary material for: Imaging evaluation of uterine arteries in potential living donors for uterus transplantation: a comparative study of MRA, CTA, and DSA
Source: Eur Radiol. 2021 Nov 12;32(4):2360–71. doi: 10.1007/s00330-021-08350-6 (PMC8921132; doi:10.1007/s00330-021-08350-6)
Supplement: Supplementary file 1 — Supplementary file1 (DOCX 103 KB) [file 330_2021_8350_MOESM1_ESM.docx]

**Supplementary figures**


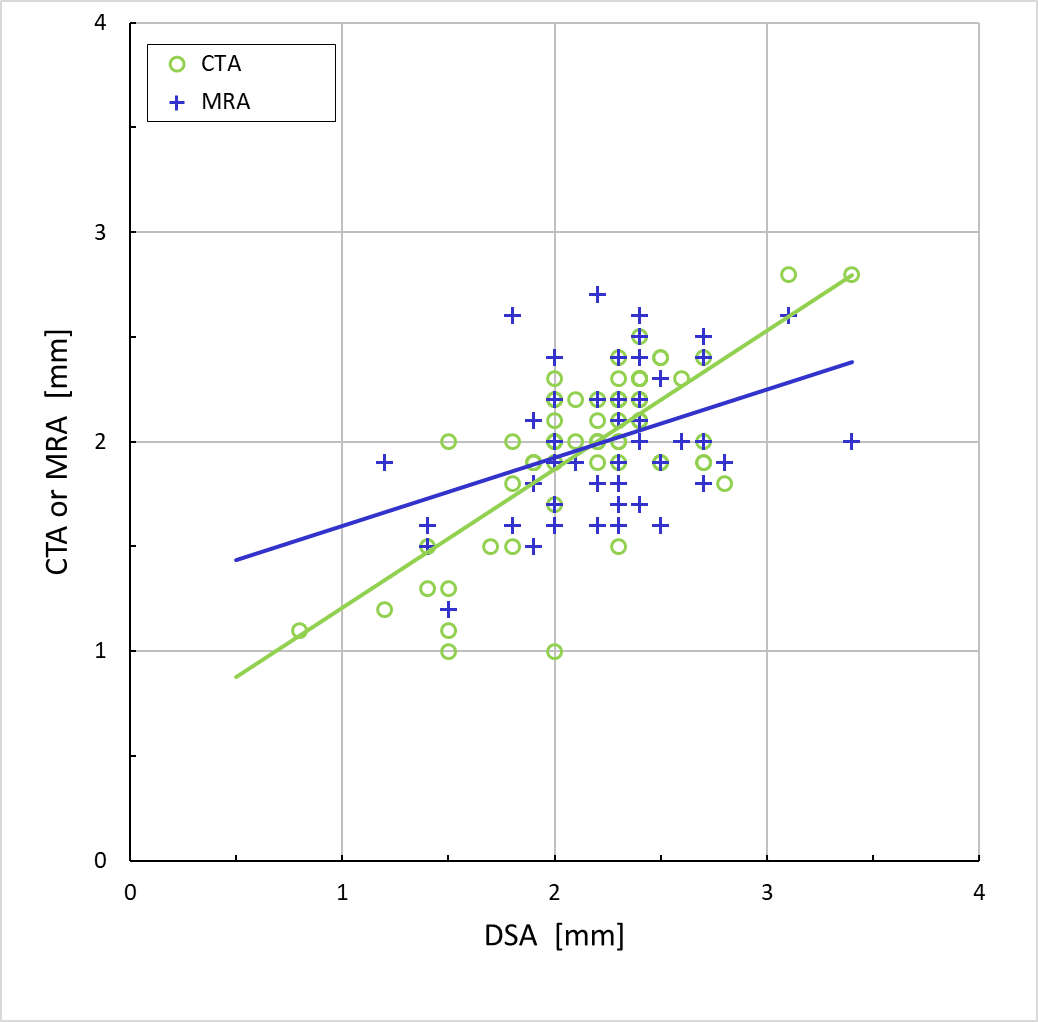


**Figure Xa.** Lumen diameter measured in CTA or MRA images compared to the corresponding measured in DSA images. The CTA and DSA examination give higher degree of agreement than MRA and DSA. CTA-DSA (Pearson’s r = +0.74) and MRA-DSA (r = +0.38).


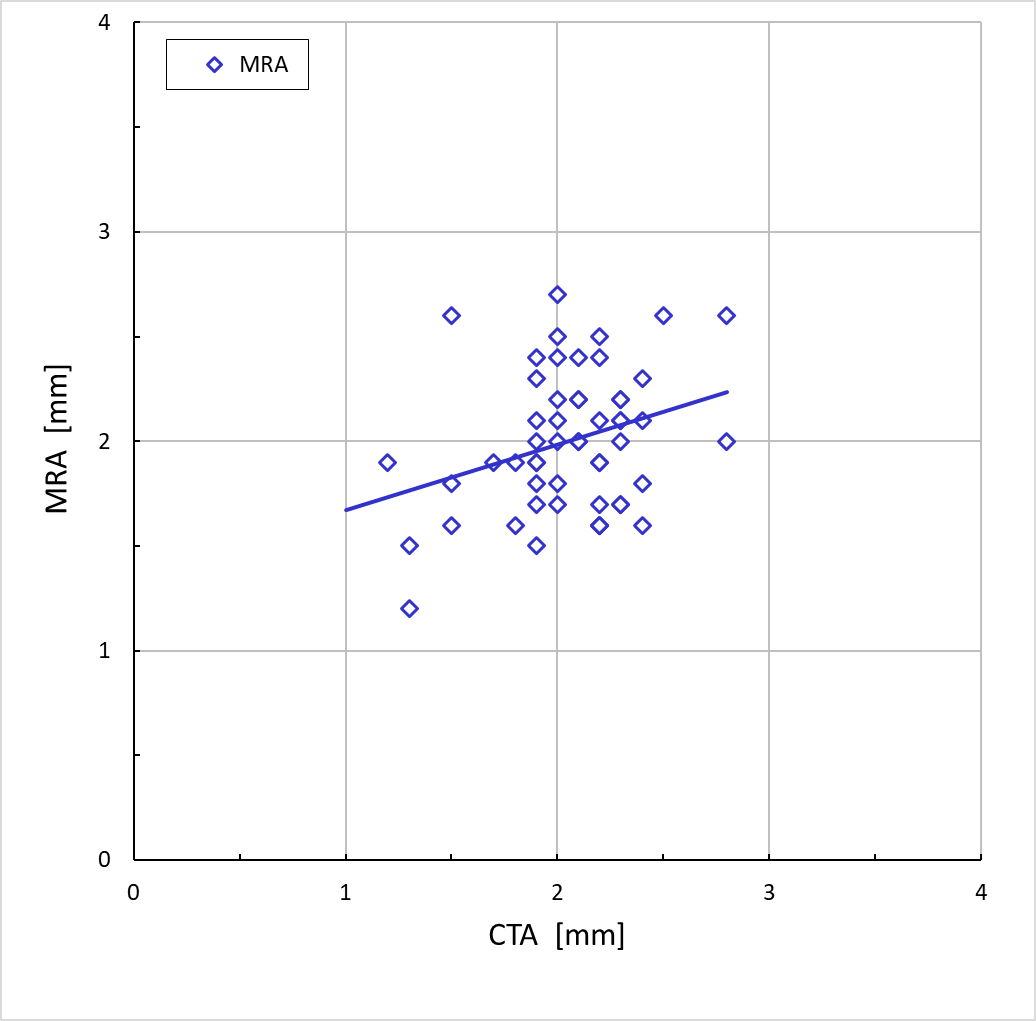


**Figure Xb.** Lumen diameter measured in MRA images compared to the corresponding measured in CTA images. MRA-CTA (Pearson’s r = +0.31).

Abbreviations: CTA=Computed Tomography Angiography and MRA=Magnetic Resonance Angiography.
